# Supplementary material for: Structure and mechanism of a phage-encoded SAM lyase revises catalytic function of enzyme family
Source: eLife. 2021 Feb 10;10:e61818. doi: 10.7554/eLife.61818 (PMC7877911; doi:10.7554/eLife.61818)
Supplement: Supplementary file 1. [file elife-61818-supp1.docx]

**Supplementary file 1**

**Structure and mechanism of a phage-encoded SAM lyase revises catalytic function of enzyme family**

Xiaohu Guo^1,5#^, Annika Söderholm^1#^, Sandesh Kanchugal P^1#^, Geir Villy Isaksen^1,2#^, Omar Warsi^3^, Ulrich Eckhard^1,6^_,_ Silvia Trigüis^1^, Adolf Gogoll^4^, Jon Jerlström-Hultqvist^3,1^, Johan Åqvist^1^, Dan I. Andersson^3^ & Maria Selmer^1*^

^1^ Department of Cell and Molecular Biology, Uppsala University, BMC, Box 596, 751 24 Uppsala, Sweden

^2^ Hylleraas Centre for Quantum Molecular Sciences, Department of Chemistry, UiT - The Arctic University of Norway, N9037, Tromsø, Norway.

^3^ Department of Medical Biochemistry and Microbiology, Uppsala University, BMC, Box 582, 751 23 Uppsala, Sweden

^4^ Department of Chemistry-BMC, Uppsala University, BMC, Box 576, 75123, Uppsala, Sweden

^5^ Current affiliation: Division of Biochemistry, Cancer Genomics Center, Netherlands Cancer Institute, Amsterdam, the Netherlands

^6^ Current affiliation: Proteolysis Lab, Department of Structural Biology, Molecular Biology Institute of Barcelona, CSIC, Barcelona Science Park, Baldiri Reixac, 15-21, 08028 Barcelona, Catalonia, Spain.

^#^ These authors contributed equally to this study.

* to whom correspondence should be addressed: maria.selmer@icm.uu.se

**Content:**

**Supplementary tables**

**Supplementary file 1-table 1:** Sequences of Svi3-3 constructs in this study

| Protein | Length (amino acids) | Sequence |
| --- | --- | --- |
| Svi3-3_d19 | 165 | MSGSHHHHHHGSSGENLYFQSLMERLGGGGFSARIFVGLNVGDKPTYTIE  DVVKDTIAIKRQGILPDASFVAQRGVYTEQRSGQLVTENSVQIIIIDLEG  LSKEDFTGKVQALGKELREDFKQESVIVEIQERGIVQDVYSITAEWYEEG  PMRPLRVDLQPSLIS |
| Svi3-3_d19 after TEV cleavage | 146 | SLMERLGGGGFSARIFVGLNVGDKPTYTIEDVVKDTIAIRKRQGILPDAS  FVAQRGVYTEQRSGQLVTENSVQIIIIDLEGLSKEDFTGKVQALGKELRE  DFKQESVIVEIQERGIVQDVYSITAEWYEEGPMRPLRVDLQPSLIS |

**Supplementary file 1-table 2**: *In vivo* complementation assay using different variants of T3 SAMase, present on the chromosome

| Strain | Genotype | M9 minimal media + Arabinose (%) | | | |
| --- | --- | --- | --- | --- | --- |
|  |  | 0 | 0.01 | 0.05 | 0.1 |
| DA5438 | wild-type | growth | growth | growth | growth |
| DA41453 | *ΔilvA* | no growth | no growth | no growth | no growth |
| DA67469 | *ΔilvA*+ T3S | no growth | growth | growth | growth |
| DA67467 | *ΔilvA*+ T3S E67Q | no growth | no growth | growth | growth |
| DA67468 | *ΔilvA* +T3S E68Q | no growth | no growth | no growth | growth |

**Supplementary file 1-table 3:** Primer sequences

| **Primer name** | **Sequence** |
| --- | --- |
| Svi3-3_d19f | ATGGAACGTCTCGGCGGCGG |
| Svi3-3_r1 | CTCAACTAATTAAGCTTGGCTGCAGG |
| Svi3-3_Y58F_f | GCTGCTCGGTGAAGACGCCGCGC |
| Svi3-3_Y58F_r | GCGCGGCGTCTTCACCGAGCAGC |
| Svi3-3_E69A_f | ACAGCTCGTCACGGCGAACTCGGTCCAGATCATC |
| Svi3-3_E69A_r | GATGATCTGGACCGAGTTCGCCGTGACGAGCTGT |
| Svi3-3_E105Q_f | CCACGATGACGCTCTGCTGCTTGAAATCCTC |
| Svi3-3_E105Q_r | GAGGATTTCAAGCAGCAGAGCGTCATCGTGG |
| araBAD_cat_sacB_F | AGTATAGCCTGGTTTCGTTTGATTGGCTGTGGTTTTATACAGTCAGTGTAGGCTGGAGCTGCTTC |
| araBAD_cat_sacB_R | TCTCTACTGTTTCTCCATACCCGTTTTTTTGGATGGAGTGAAACGCATATGAATATCCTCCTTAGTTCC |
| T3SAM_int_catsacBF | CGTGCCGCACCGGGTCTTTATGGCTCCGTTGAGTCAACCGATTTG GTGTAGGCTGGAGCTGCTTC |
| T3SAM_int_catsacBR | TTCCTCAGTTGGTGCGCTTGAGATTGCCTCACGATAGCACCCGGTCATATGAATATCCTCCTTAGTTCC |
| ara_t3_SAMF | TCTCTACTGTTTCTCCATACCCGTTTTTTTGGATGGAGTGAAACGATGATTTTCACTAAAGAGCCTG |
| ara_t3_SAMR | AGTATAGCCTGGTTTCGTTTGATTGGCTGTGGTTTTATACAGTCA TTATTGTACTTGCCAGCGGCGACC |
| Test_primer_f | ACCCCGCTTATTAAAAGCAT |
| Test_primer_r | AAATCCATCAAAAAACCAGG |
